# Patients: From the Doctor's Point of View

**Published:** 1887-07-02

**Authors:** 


### PATIENTS: FROM THE DOCTOR'S POINT OF VIEW.

By a Consulting Physician.

"Oh wad some pow'r the giftie gie us,
To see oursels as others see us!
It wad frae monie a blunder free us,
And foolish notion."

Burns was neither spiteful nor cynical, but, like other men who have lived in the world with their eyes open, he had seen a good many things in his time which made him think some human beings very small persons. The inside of the doctor's consulting-room could probably tell a more varied story of human life than almost any other space enclosed by four walls in the whole world. But the story would by no means be such as cynics and scoffers expect. Weaknesses it might record; follies and even sins of many and diverse kinds would form part of it. But on the whole the story would be one of duty attempted by hands and brains enfeebled by disease; of sufferings endured with martyr-like patience for the sake of husband and children; of self-denial and devotion to the little world of home which well deserve to be called "heroic."

The successful doctor is often said by the envious to be "an old woman." The family physician should have a great deal of the woman in him; not perhaps necessarily of the "old woman," but at any rate of the middle aged, patient, large-hearted, motherly woman who has learnt the arts of patience and sympathy in the best of all schools. For what, after all, is the tried and trusted family doctor? Is he not the high priest of the nursery, the father confessor of the family circle, the wife's constant counsellor, and the husband's honoured friend? It is to be feared that this old-fashioned view of "the doctor" is getting a little out of date, and especially in London, where specialists and beardless consultants are as thick as blackberries in October. But where the family attendant is a competent, sympathetic, and conscientious man, the old-fashioned relations between doctor and patient are gradually established even in this overgrown metropolis. The family doctor of the right kind is really the friend of the family. If he be both conscientious and courageous, he can be of inestimable service in an infinite variety of ways. But he is not always courageous, though conscientious we may trust he is; and this is the reason why the present writer wishes to say a few words, more especially to lady patients, which the doctor would often like to say, but fears to do so. For, ladies, you must remember that the doctor is seldom a man of means: he has no opportunities of accumulating resources except by the smallest and most miserly savings. His money is earned by himself personally, not by clerks and shopmen who take their instructions from him. It is earned, not merely by the sweat of his brow, but by the sweat of his brain and of his heart. The true doctor's life is an unceasing struggle, not only for the maintenance of his own health and home, but for the preservation of the health and life, and peace of mind of every family under his care. It should be remembered that the doctor often wishes to be plain spoken and candid with his patients, but dare not. He thinks of his wife and children—those hostages to fortune—and permits Mr. Smith to have two glasses of grog in the day, when one or none would be better for him. For the same reason he refrains from remonstrating with Mrs. Robinson when she wears low-necked dresses, tight corsets, high-heeled boots, and every other ridiculous and mad abomination which modern fashion dictates. But it is a mistake for patients to put a muzzle on the doctor's mouth. Many a weary illness would be avoided, many a period of domestic misrule prevented, many a ten-pound note kept in the pocket, if he were encouraged to be frank and outspoken.

On the other hand, patients, and especially lady patients, are sometimes morbidly curious to know the exact nature, the probable duration, the symptoms to be expected, and the whole history of the case down to its minutest details. They do not then wish for the doctor to be silent, but to speak, and to speak with the utmost precision and fulness. Well, the curiosity is not unnatural, and if the doctor respond to it only by a wise shake of the head and an elaborate silence, it may be feared that his knowledge is not so great as could be wished. But too much curiosity on the part of patients and friends is not always a good thing. Sometimes it forces the doctor into making statements with confident authority, which all the time he knows to be only guesses. There are men, who having once advanced an opinion, think it a sign of incompetence to change it, and so stick to it through thick and thin, although the facts may be all obviously against them. Of course this is wrong. It is as foolish as it is cowardly and immoral. But the patients are often quite as much to be blamed as the doctor: they insist upon being told something definite, and so the doctor, to save his credit as he thinks, tells them something definite which will happen on occasion to be definitely and decidedly wrong. This is the thing to do: If you have an honest and capable doctor, give him time; let him announce his judgment when it is ripe. There are cases, and many of them too, where the whole College of Physicians could not say what the disease is or what it is going to be. Is this strange? Consider the difficulties of the work: think where the diseased processes are—out of sight almost always, often absolutely beyond the reach of eye, of ear, of finger, of stethoscope, of thermometer, of any and every method and instrument which ancient or modern science has been able to discover or invent. To the outsider doctors no doubt often appear ignorant and incapable men: to the initiated they are often absolute miracles of painstaking observation, accurate reasoning, and experienced skill.

Of course it must be admitted that there are bad doctors, ignorant, knavish, unscrupulous, incompetent, and worthless scoundrels: men who are fitter for the prisoner's cell than for the sanctities of the domestic hearth. But, take the word of experience for it, these are the few. The average family doctor is a man of honourable character and good intentions. If he be trusted he will endeavour to deserve still greater trust. Trust him, and you take away one of his principal temptations to appear infallible, and therefore to be dishonest. The best relationship which can subsist between doctor and family is that of tried and trusted friendship. If this be the relation sustained towards the family doctor, it should be preserved sacred and inviolate so long as his character is such as to make it possible and desirable.

